# Atenolol Ameliorates Skeletal Muscle Atrophy and Oxidative Stress Induced by Cast Immobilization in Rats

**DOI:** 10.3390/biomedicines11051269

**Published:** 2023-04-25

**Authors:** Anand Kumar, Chaitany Jayprakash Raorane, Deepak Rawat, Priyanka Prajapati, Ritu Raj, Dinesh Kumar, Seong-Cheol Kim, Vinit Raj, Sapana Kushwaha

**Affiliations:** 1 Department of Pharmaceutical Sciences, School of Pharmaceutical Sciences, Babasaheb Bhimrao Ambedkar University, Vidya Vihar, Raebareli Road, Lucknow 226025, India; anandkumarpharm@gmail.com (A.K.); deepakrawat4287@gmail.com (D.R.); priyankaprajapati243@gmail.com (P.P.); 2 School of Chemical Engineering, Yeungnam University, Gyeongsan 38541, Republic of Korea; chaitanyaraorane22@ynu.ac.kr (C.J.R.); sckim07@ynu.ac.kr (S.-C.K.); 3 Centre of Biomedical Research, SGPGIMS Campus, Lucknow 226014, India; riturajbio444@gmail.com (R.R.); dineshcbmr@gmail.com (D.K.); 4 National Institutes of Pharmaceutical Education and Research, Raebareli (NIPER-R), New Transit Campus, Bijnor-Sisendi Road, Lucknow 226002, India

**Keywords:** cast immobilization, atenolol, oxidative stress, metabolomics, skeletal muscle atrophy

## Abstract

(1) Background: Skeletal muscle atrophy is a common and debilitating condition associated with disease, bed rest, and inactivity. We aimed to investigate the effect of atenolol (ATN) on cast immobilization (IM)-induced skeletal muscle loss. (2) Methods: Eighteen male albino Wistar rats were divided into three groups: a control group, an IM group (14 days), and an IM+ATN group (10 mg/kg, orally for 14 days). After the last dose of atenolol, forced swimming test, rotarod test, and footprint analysis were performed, and skeletal muscle loss was determined. Animals were then sacrificed. Serum and gastrocnemius (GN) muscles were then collected, serum creatinine, GN muscle antioxidant, and oxidative stress levels were determined, and histopathology and ^1^H NMR profiling of serum metabolites were performed. (3) Results: Atenolol significantly prevented immobilization-induced changes in creatinine, antioxidant, and oxidative stress levels. Furthermore, GN muscle histology results showed that atenolol significantly increased cross-sectional muscle area and Feret’s diameter. Metabolomics profiling showed that glutamine-to-glucose ratio and pyruvate, succinate, valine, citrate, leucine, isoleucine, phenylalanine, acetone, serine, and 3-hydroxybutyrate levels were significantly higher, that alanine and proline levels were significantly lower in the IM group than in the control group, and that atenolol administration suppressed these metabolite changes. (4) Conclusions: Atenolol reduced immobilization-induced skeletal muscle wasting and might protect against the deleterious effects of prolonged bed rest.

## 1. Introduction

Skeletal muscle atrophy is defined as a loss of skeletal muscle mass and may occur as a consequence of diseases such as cancer, acquired immunodeficiency syndrome, sepsis, burn injury, organ failure, or respiratory or metabolic disease [1]. Disuse atrophy (also called immobilization) is defined as a loss of skeletal muscle mass due to inactivity or lower activity than normal and usually affects a single group of muscles. This condition usually occurs after prolonged bed rest, spinal cord injury, exposure to microgravity, or intensive care unit (ICU) stay and is commonly encountered after cast application for fracture management or permanent bed rest [2]. On the other hand, neuronal muscle atrophy occurs after denervation or spinal cord injury. Studies have shown that astronauts exposed to microgravity suffer considerable bone strength and cross-sectional muscle area and volume losses [3] and that ~50% of ICU patients under mechanical ventilation exhibit signs of muscle atrophy [2]. Furthermore, studies have demonstrated that immobilized muscle exhibits a catabolic condition as evidenced by accelerated reductions in body weight and muscle mass and reduced muscle fiber sizes and numbers [4,5]. In addition, prolonged immobilization leads to mitochondrial structural, biogenetic, and functional malfunctions, which exacerbate skeletal muscle deterioration [6]. A number of investigations indicate that muscle immobilization increases proteolysis and inflammatory signaling and oxidative stress and alters metabolic functions [7]. Metabolomics has been used recently to identify metabolic changes in muscles and muscle-associated diseases [8], and the findings obtained show that abnormal glutamic acid levels and associated amino acid metabolic changes may be indicative of impaired energy metabolism pathways in skeletal muscle [9]. However, few metabolomic studies have addressed the effects of cast immobilization. One study showed that 7 days of bed rest led to a significant decrease in the activities of citrate synthase and β–hydroxyacyl-coA dehydrogenase, indicating loss of mitochondrial mass, and that 14 days of bed rest reduced mitochondrial biogenesis and oxidative metabolism [10].

The primary strategies used to treat muscle wasting include exercise, nutrition, acupuncture, hormonal therapies, and pharmacological intervention with angiotensin receptor blockers, angiotensin-converting enzyme blockers, or β2-adrenoceptor agonists [11]. At the time of writing, nusinersen (SPINRAZA) and Zolgensma were approved by the Food and Drug Administration (FDA) for treating spinal muscle atrophy (SMA) in children less than two years old [12], and recently, the FDA approved risdiplam (Evrysdi) as the first oral treatment for SMA [13]. Thus, there is an urgent need to identify new or existing therapeutics capable of treating muscle wasting and its associated diseases. Atenolol (a cardio-selective β1-blocker) is widely used to treat cardiovascular diseases such as congestive heart failure, hypertension, and angina pectoris, whereas β-blockers are used to treat non-cardiac conditions such as glaucoma, migraine, anxiety, tremors, and thyrotoxicosis [14].

Recently, researchers reported that beta-blocker therapy might be suitable for massive burns [15,16], which suggests beta-blockers might also promote muscle mass recovery in critically ill patients. Furthermore, prolonged treatment of B6D2F1 male mice with atenolol (0.1 g L^−1^ in drinking water) reduced glyco-oxidative and mitochondrial protein oxidative damage significantly in cardiac and skeletal muscle, and improved immune and behavioral functions such as motor coordination and muscle strength. In addition, in the same study, atenolol significantly increased the level of complex II in heart mitochondria and p-ERK and total ERK levels in heart and skeletal muscles in old mice as compared with age-matched controls [17]. These findings indicate that atenolol prevented aging-related detrimental changes. Moreover, atenolol improved cardiac function, reduced cardiac fibrosis, and restored cardiomyocytes in the transverse aortic constriction (TAC) heart failure model [18]. In addition, it showed myocyte-enhanced factor 2 (MEF2) is the critical transcription factor that plays a crucial role in regulating cardiac, skeletal, and smooth muscle differentiation [18]. Taken together, the above findings showed that atenolol is able to protect cardiac and skeletal muscle in disease conditions. Contrarily, atenolol was reported to reduce exercise endurance capacity [19] and loss of muscle function in older women after taking the drug for <1 month [20,21]. Therefore, in the present study, we investigated the effect of atenolol on cast-immobilization-induced skeletal muscle wasting in Wistar rats and performed serum metabolomics analysis using ^1^H-NMR to investigate metabolic changes in immobilized rats and to determine whether these changes are influenced by atenolol.

## 2. Materials and Methods

### 2.1. Chemicals

Atenolol was purchased from Sigma-Aldrich (St. Louis, MO, USA). Delta-Cast^®^ Elite was purchased from BSN Medical (an Essity Company, Hamburg, Germany). Chemicals were purchased from MP Biomedicals and Sigma-Aldrich, and Creatinine kits were purchased from Proton Biochemicals Pvt. Ltd. Bengaluru, India). All solvents used were of analytical grade (99% purity).

### 2.2. Animal Allocations and Ethical Approval

Male albino Wistar rats (130 ± 10 g) were used in the study. Animals were acclimatized to laboratory conditions for two weeks before experiments and housed in cages under controlled conditions (23 ± 2 °C, 12 h light/dark cycle) with free access to a standard pellet diet and water. The experiment was performed in accord with the guidelines issued by the Committee for the Purpose of Control and Supervision of Experiments on Animals (CPCSEA) guidelines for laboratory animals, and its ethical status was approved by our institutional ethics committee (IAEC no. BBDNIIT/IAEC/2019/10).

### 2.3. Experimental Design

Eighteen animals were randomly divided into three groups (n = 6), as follows; the control group that was not immobilized, the immobilized (IM) group, or the IM+ATN group. Atenolol (ATN) was freshly prepared in normal saline and administered orally at 10 mg/kg for 14 consecutive days; the dose was derived from the literature [22]. After the last dose of atenolol, animals were subjected to rotarod and forced swimming tests, and footprint analysis was performed to assess muscle coordination. At the end of the experiment, animals were sacrificed by cervical dislocation under mild anesthesia. Serum was collected, and gastrocnemius (GN) muscles were isolated for histopathology and endpoint parameters.

#### Hind Limb Cast-Immobilization-Induced Skeletal Muscle Atrophy

One hind limb was immobilized in plantar flexion using a Delta-Cast^®^ ElitePlaster Cast applied from the trunk to the middle of the left hind leg under mild anesthesia. Contralateral limbs served as treatment-naïve. Plaster casts were replaced when any sign of circulation impairment (e.g., congestion, ischemia, or ulcer formation) was observed or a cast was damaged during the 14-day experimental period, as previously described [23].

### 2.4. Body and Gastrocnemius Muscle Weights

Body weights were measured before and after 14 days of ATN treatment. On day 15, rats were sacrificed, and GN muscles were weighed.

### 2.5. Assessment of Muscle Function and Coordination Tests

#### 2.5.1. Rotarod Test

A rotarod test was used to evaluate motor coordination and grip strength. Briefly, after acclimatization for three consecutive days, rats were placed on top of a rod on the rotating beam (25–40 rpm). Next, retention times on the rod were recorded and analyzed as previously described [24].

#### 2.5.2. Footprint Pattern Analysis

Footprint pattern analysis was used to evaluate walking stability and body balance. Briefly, hind feet were stained with blue ink, and rats were trained to walk on a blank sheet of paper in a corridor (100 cm long, 10 cm wide, and 20 cm high) made of cardboard for 3 days before the experiment; the process was repeated when a rat did not walk the full test length. Paper sheets were air-dried, and step (stride) lengths (cm) and widths (cm) were determined as previously described [24].

#### 2.5.3. Forced Swimming Test

A forced swimming test was used to validate the cast-induced immobilization model and assess skeletal muscle function. In brief, a rat was placed in a glass cylinder (40 cm high, 18 cm in diameter) containing 25 cm of water at 25 °C. Times spent swimming and floating over 90 s were recorded, and data were analyzed as previously described [25].

### 2.6. Biochemical Measurements

#### 2.6.1. Creatinine Levels

Muscle damage was assessed by measuring serum creatinine levels using the creatinine kit.

#### 2.6.2. Estimation of Oxidative Stress and Antioxidant Levels

##### Analysis of MDA (Malondialdehyde) Levels in Tissue Samples

MDA levels were measured as a surrogate of lipid peroxidation. Briefly, 0.5 mL of 30% trichloroacetic acid (TCA) and 0.5 mL of 0.8% thiobarbituric acid (TBA) were added to 100 mg of gastrocnemius (GN) tissue homogenate, placed in a shaking water bath for 30 min at 80 °C, cooled for 15 min, and centrifuged at 3000 rpm for 15 min. Absorbances were measured at 540 nm against a blank. As an MDA standard, 1, 1, 3, 3 tetraethoxy propane was used. Lipid peroxidation was calculated from a standard curve and expressed as nM MDA/g of protein [26].

##### Estimation of Glutathione (GSH)

GN tissue homogenate (125 µL, 10%) was prepared in phosphate buffer. Totals of 100 µL of distilled water and 25 µL of 50% TCA were added, vortexed for 10 min, and centrifuged at 5000 rpm for 10 min. Tris buffer (131 µL, pH-8.0) and Ellman’s reagent (3 µL) were then added to 66 µL aliquots of supernatants, and absorbances were measured at 405 nm [26].

##### Estimation of Superoxide Dismutase (SOD)

A total of 100 µL of gastrocnemius tissue homogenate was added to tris HCl buffer (pH 8.5), and the final volume was adjusted to 3 mL using the same buffer. Pyrogallol (25 µL) was then added, and absorbances were recorded at 420 nm for 3 min at 1 min intervals. Blanks were prepared without homogenate. SOD concentrations were determined using a pyrogallol standard curve by measuring absorbance at 420 nm, and results are expressed as U/µg of protein [26].

### 2.7. Estimation of Myofibrillar Protein Contents

Myofibrillar protein contents of GN muscles were analyzed using Lowry’s method. In brief, GN muscles were quickly removed, rinsed in cold saline, dried on filter paper, and weighed. GN muscle (100 mg) was then homogenized in 5% ice-cold buffer containing 0.25 M sucrose, 2 mM EDTA, and 10 mM Tris–HCl (pH 7.4) and centrifuged at 600× *g*. Supernatants were discarded, and pellets were suspended in 0.5 M KCl. Protein contents were measured using Lowry’s method using bovine serum albumin (BSA) as the standard [27].

### 2.8. Estimation of Cellular Damage by Histology

Histopathological studies were performed by hematoxylin and eosin (H&E) staining to observe morphological changes in GN muscles. Briefly, GN muscles were fixed in 10% formalin solution, dehydrated using an ethanol series (70, 90, and 100%) and xylene, embedded in paraffin wax, and sectioned at 5 μm using a microtome. Sections were stained then with H&E and observed under a microscope equipped with a digital imaging system [27]. Cross-sectional areas and Feret’s diameters of GN muscles were determined using 15–20 random microscopic fields (5 fields/animal, 3 animals/group). Quantification was performed using Image J software (NIH, USA) [28].

### 2.9. ^1^H Nuclear Magnetic Resonance (NMR)-Based Serum Metabolic Profiling

#### 2.9.1. Sample Preparation

A total of 250 µL serum sample was added with 250 µL 100% deuterium oxide (D_2_O). Sample were then centrifuged at 16,278× *g* for 5 min. A total of 450 µL supernatants were subjected to ^1^H NMR, 5 mm NMR tubes (Wilmad Glass, USA) in tightly sealed capillary tubes, and the sodium salt of -trimethylsilyl-(2,2,3,3-d4)-propionic acid (TSP) was used as a reference. D_2_O was served as a co-solvent and a source of a deuterium field/frequency lock [29].

#### 2.9.2. NMR Measurements

^1^H NMR was performed at 298 K using an 800 MHz NMR spectrometer (AVANCE-III, equipped with a Cryoprobe). The serum metabolic profiles were obtained using a one-dimensional (1D) Carr–Purcell–Meiboom–Gill pulse sequence (^1^H CPMG) NMR. Results were recorded using the “cpmgpr1d” pulse program (Bruker standard library), and signals was pre-saturated with a water signal having a “recycle delay” (RD) of 5 s. Acquisition parameters were used: 12 ppm, width of spectral sweep; 32,768 data points were used; 5 s time of total relaxation delay (RD); 128 transient numbers, and T2 filtering (suppressing broad peaks of macromolecules) was acquired with an echo time of 200 µs reiterated 300 times, which includes the complete duration of effective echo time (60 ms). NMR spectra were processed using Bruker NMR data Processing Software Topspin (v2.1) software (Bruker BioSpin GmbH, Rheinstetten, Germany) using a typical Fourier transformation (FT) approach as the primary processing step. However, before performing FT, each free induction decay (FID) was zero-filled to a total of 65,536 data points and then multiplied by an exponential line broadening function with a frequency of 0.3 Hz [29].

#### 2.9.3. Spectral Assignments and Concentration Profiling

All peaks in the ^1^D and ^1^H CPMG NMR spectra were identified and annotated for different serum metabolites using Chenomx NMR suite’s 800 MHz compound spectral database library at pH 7.2 for all samples (Chenomx Inc., Edmonton, AB, Canada). 2D homonuclear ^1^H-^1^H TOCSY (total correlation spectroscopy) and heteronuclear ^1^H-^13^C HSQC (heteronuclear single quantum coherence) NMR peaks were also used to aid identification and annotation [29]. Assignments were confirmed using publicly accessible databases (BMRB: www.bmrb.wisc.edu/metabolomics (accessed on 15 September 2022)) and HMBD: http://www.hmdb.ca/(accessed on 21 October 2022)) and previously published NMR assignments of metabolites [29]. All CPMG pulse NMR spectra collected were visually inspected to confirm acceptability. The NMR suite of CHENOMX software was used for additional analysis (Chenomx Inc., Edmonton, AB, Canada). All NMR spectra were first baseline corrected and calibrated internally versus the ^1^H NMR peak of formate (at = 8.43 ppm and 0.01 mM). Concentration profiling of the following 37 serum metabolites was conducted; 3-hydroxy-butyrate (3HB), acetate, acetone, alanine, asparagine, betaine, choline, citrate, creatine, dimethyl-sulfone (DMS), dimethyl-amine (DMA), glutamine, glycerol, glycine, isobutyrate (IsoB), glucose, glutamate, isoleucine, lactate, leucine, methanol, pyruvate, serine, succinate, threonine, tyrosine, phenylalanine, proline, valine, myo-inositol, and histidine. Metabolic characteristics were then employed to evaluate five significant metabolic ratios: branched-chain amino acid-to-tyrosine ratio (BTR), histidine-to-tyrosine ratio (HTR), phenylalanine-to-tyrosine ratio (PTR), lactate-to-pyruvate ratio (LPR), and glutamine-to-glucose ratio (QGR) [30].

#### 2.9.4. Multivariate Data Analysis

Metabolite concentrations and ratios were transferred to Microsoft Office (MS) Excel, annotated for sample class, and transformed into comma-separated value (CSV) text format. A CSV file was used for multivariate statistical data analysis using modules MetaboAnalyst 5.0 (https://www.metaboanalyst.ca (accessed on 21 October 2022)) [30]. Initial evaluations of metabolites in the dataset and the identification of outliers were achieved by (PCA) principal component analysis. Supervised (PLS-DA) partial least squares discriminant analysis was used to identify metabolites responsible for group separations and the group trend and outlier detection. In addition, a 10-fold cross-validation procedure was used to prevent the PLS-DA model from being closely fitted to the data. NMR spectra were processed using the PROFILER-Module of CHENOMX to determine serum metabolic profiles. Additionally, the serum concentrations of specific metabolites were calculated for the control, IM, and IM+ATN groups. The resulting serum metabolic profiles of the three groups were compared by PLS-DA analysis. The qualities of PLS-DA models were evaluated by permutation analysis using cross-validation testing one hundred times. Results were used to calculate R^2^ values (the goodness-of-fit parameter) and Q^2^ values (the goodness-of-prediction parameter), respectively. Variable importance on projection (VIP) scores having a value of >1.0 were used. The PLS-DA model was utilized to identify the metabolites responsible for the discrimination of different experimental groups. Chenomx NMR Suite v8.1 (Chenomx Inc., Edmonton, AB, Canada) was used to estimate the serum concentrations of critical metabolites [31].

### 2.10. Statistical Analysis

Data were analyzed using GraphPad Prism software (version 8.01) (Founded by Dr. Harvey Motulsky Boston, MA, USA). The normality of variable distributions was first tested using the Shapiro–Wilk test, and homoscedasticity (Brown–Forsythe test) was used to test the equality of group variables. Results are expressed as mean ± standard deviation (SD). Further, the analysis was conducted using one-way analysis of variance (ANOVA) followed by Tukey’s *post hoc* test for multiple comparisons. Statistical significance was accepted for *p* values < 0.05.

## 3. Results

### 3.1. Effects of Atenolol on Body Weight and Gastrocnemius (GN) Muscle Weight in Immobilized Rat

**Figure 1:**
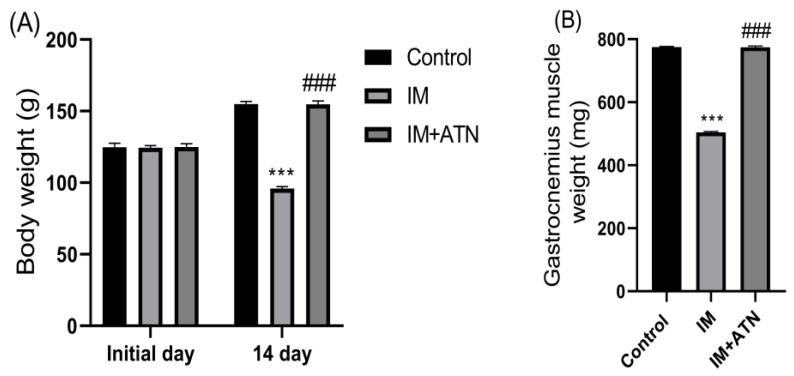
Effect of atenolol on (**A**) Body weight (g) over the 14-day experimental period and (**B**) Gastrocnemius muscle weight (mg). Data were represented as mean ± SD (n = 6). Statistical significance was determined by using one-way ANOVA with Tukey’s multiple comparison *post hoc* test, *** *p* < 0.001 vs. control and ### *p* < 0.001 vs. IM. IM–Immobilized and ATN–Atenolol.

At the end of the study, the IM group showed significant decreases in body weight and GN muscle weight as compared with the control group. However, body weight (F_2,15_ = 1.234) and GN muscle weight (F_2,15_ = 3.457) were significantly greater in the IM+ATN group than in the IM group (Figure 1A,B).

### 3.2. Effect of Atenolol on Footprint Patterns, Rotarod, and Forced Swimming in Immobilized Rat

**Figure 2:**
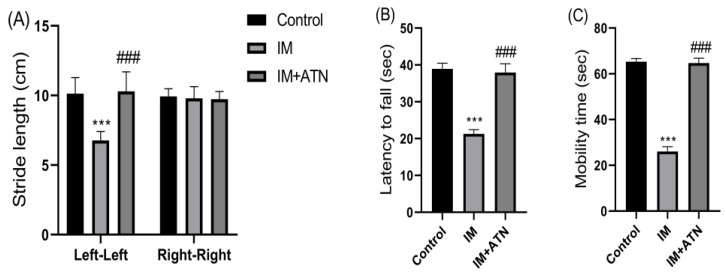
Effect of atenolol on (**A**) Footprint pattern test (stride length in cm), (**B**) Rotarod test (Latency to fall in seconds) for muscle function and coordination, and (**C**) Forced swimming test (Immobility time in seconds). Data were represented as mean ± SD (n = 6). Statistical significance was determined by using one-way ANOVA with Tukey’s multiple comparison *post hoc* test, *** p < 0.001 vs. control and *### p <* 0.001 vs. IM. IM–Immobilized and ATN—Atenolol.

Footprint pattern analysis, rotarod, and forced swimming tests were conducted after 14 days. It provides an excellent means of assessing walking stability and body balance. Footprint analysis results showed that atenolol significantly increased stride length (gait) left-to-left (F_2,15_ = 0.4358) and right-to-right (F_2,15_ = 0.7072) in the IM+ATN group versus the IM group (Figure 2A). Rotarod results showed that IM significantly reduced muscle coordination and that atenolol significantly attenuated this effect of IM (F_2,15_ = 1.450) (Figure 2B). Furthermore, the forced swimming test showed that mobility times were significantly greater in the IM+ATN group than in the IM group (F_2,15_ = 0.6957) (Figure 2C).

### 3.3. Effect of Atenolol on Myofibrillar (Gastrocnemius Muscle) Protein Contents in Immobilized Rat

**Figure 3:**
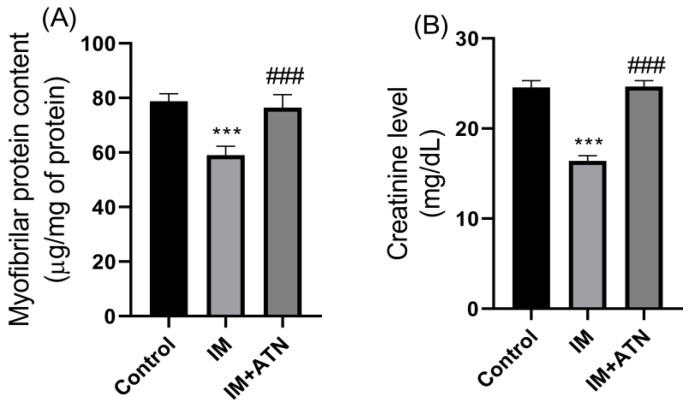
Effect of atenolol on (**A**) Myofibrillar protein content (µg/mg of protein) and (**B**) Serum creatinine level (mg/dL). Data were represented as mean ± SD (n = 6). Statistical significance was determined using one-way ANOVA with Tukey’s multiple comparison *post hoc* test, *** *p* < 0.001 vs. control and ### *p* < 0.001 vs. IM. IM–Immobilized and ATN—Atenolol.

Myofibrillar protein content reflects the extent of muscle proteolysis. Results showed that GN muscles in the IM group exhibited significant muscle protein degradation (F_2,15_ = 1.290) as compared to control muscles and that atenolol significantly increased myofibrillar protein contents in immobilized GN muscles (Figure 3A).

### 3.4. Effect of Atenolol on Serum Creatinine Levels in Immobilized Rats

Serum creatinine is used as a surrogate marker of muscle mass. Results showed that serum creatinine (F_2,15_ = 0.4054) levels were significantly higher in the IM+ATN group than in the IM group (Figure 3B).

### 3.5. Effect of Atenolol on Markers of Antioxidant and Oxidative Stress Levels in Immobilized Rats

**Figure 4:**
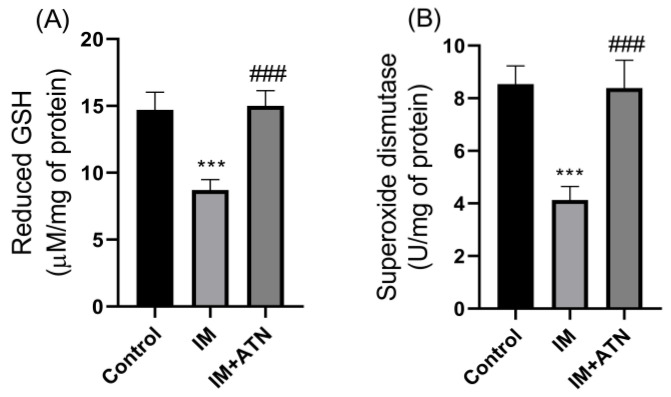

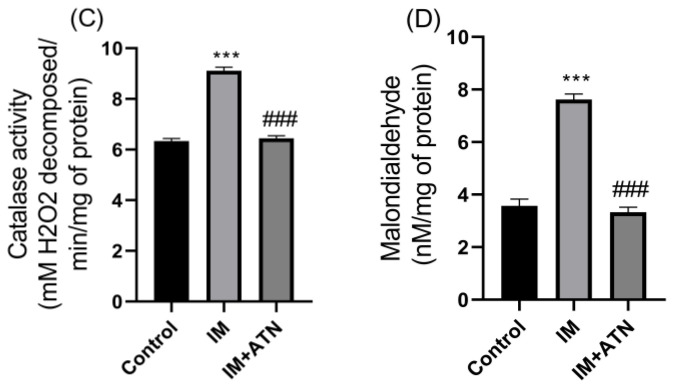
Effect of atenolol on antioxidants and oxidative stress levels (**A**) Reduced GSH (µM/mg of protein), (**B**) SOD (U/mg of protein), (**C**) Catalase (mM H_2_O_2_ decomposition/min/mg of protein), and (**D**) MDA (nM/mg of protein). Data were represented as mean ± SD (n = 6). Statistical significance was determined using one-way ANOVA with Tukey’s multiple comparison *post hoc* test, *** *p* < 0.001 vs. control and ### *p* < 0.001 vs. IM. IM—Immobilized and ATN—Atenolol.

Next, antioxidant levels, *viz.* reduced glutathione (GSH), superoxide dismutase (SOD), and catalase, and malondialdehyde (MDA, a marker of oxidative stress) levels, was measured in GN muscles. Results showed that atenolol significantly suppressed IM-induced reductions in GSH (F_2,15_ = 1.084), SOD (F_2,15_ = 0.9076), and catalase (F_2,15_ = 0.2041) levels (Figure 4A–C), and IM-induced increases (F_2,15_ = 0.4100) in MDA in GN muscles (Figure 4D). Taken together, these results suggest atenolol has antioxidant properties.

### 3.6. Effect of Atenolol on Gastrocnemius Histology in Immobilized Rats

**Figure 5:**
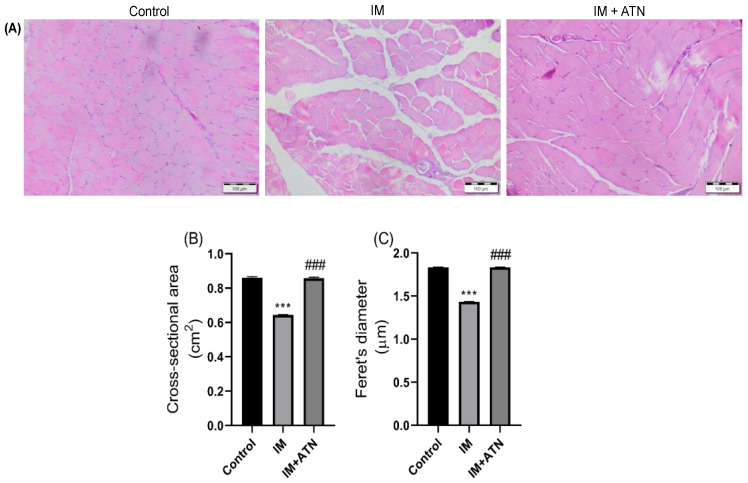
Effects of atenolol on muscle cellular architecture in the cast-immobilized (IM) rats (**A**) Representative photomicrographs of GN muscles stained by Hematoxylin and eosin (Magnification: 20×), (**B**) Cross-sectional area (cm^2^), and (**C**) Feret’s diameters (µm). Data were represented as mean ± SD (n = 6). Statistical significance was determined using one-way ANOVA with Tukey’s multiple comparison *post hoc* test, *** *p* < 0.001 vs. control and ### *p* < 0.001 vs. IM. IM—Immobilized and ATN—Atenolol.

H&E staining showed that immobilized GN muscles exhibited increased interfascicular spacings among muscle fibers and that atenolol suppressed this change and improved muscle fiber integrity as compared with the IM group (Figure 5A). Morphometric analysis showed that GN cross-sectional area (CSA) (F_2,15_ = 1.321) and Feret’s diameter (F_2,15_ = 1.376) were significantly lower in the IM group than in the control group, and that atenolol significantly inhibited these IM-induced reductions (Figure 5B,C).

### 3.7. ^1^H NMR-Based Serum Metabolomics of Muscle Wasting in Immobilized Rats

**Figure 6:**
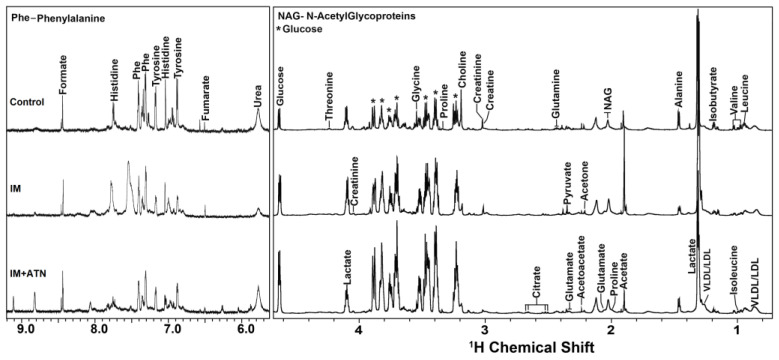
Stack plot of representative 800 MHz one-dimensional ^1^H CPMG NMR spectra of serum in the three study groups: Control, Immobilized (IM), and immobilized + atenolol (IM+ATN). The spectral peaks of specific metabolites are labelled as per their resonance assignment confirmed using annotations and determined by CHENOMX profiler and topspin. HDL: high-density lipoprotein; LDL: low-density lipoprotein; VLDL: very-low density lipoprotein; IM: Immobilized and ATN: Atenolol.

Stack plots of assigned ^1^D and ^1^H NMR spectra of rat serum samples were obtained for the control, IM, and IM+ATN groups. The NMR peaks of metabolites are annotated (Figure 6 and Tables S1 and S2), and signals were observed for abundant serum metabolites, including (a) amino acids, *viz.* alanine, glutamine, glutamate, proline, glycine, methionine, valine, leucine, isoleucine, threonine, serine, phenylalanine, tyrosine, and histidine; (b) energy metabolites, *viz.* glucose, glycerol, lactate, creatine, citrate, fumarate, formate, myo-inositol, acetate, pyruvate, and succinate; (c) lipoproteins *viz*. low-density lipoprotein (LDL) and very low-density lipoprotein (VLDL); and (d) ketone body contents, *viz.* acetone, betaine, and 3-hydroxybutyrate (3 HB). CHENOMX software was used to estimate the levels of various metabolites using formate as the internal control. Univariate and multivariate analysis tools were then used to identify metabolites of discriminatory significance. The details are provided below. In addition, concentration levels were used to estimate metabolic ratios of pathobiological relevance [32], namely, the glutamine to glucose ratio (QGR, an indicator of active inflammation [33], histidine-to-tyrosine ratio (HTR; reductions of which serve as an indicator of inflammation [32,34]), phenylalanine-to-tyrosine ratio (PTR, elevated levels of which serve as an indicator of oxidative stress [30,32]), lactate-to-pyruvate ratio (LPR, elevated levels serve as an indicator of hypoxia [32,35]), and branched-chain amino acid to tyrosine ratio (BTR).

**Figure 7:**
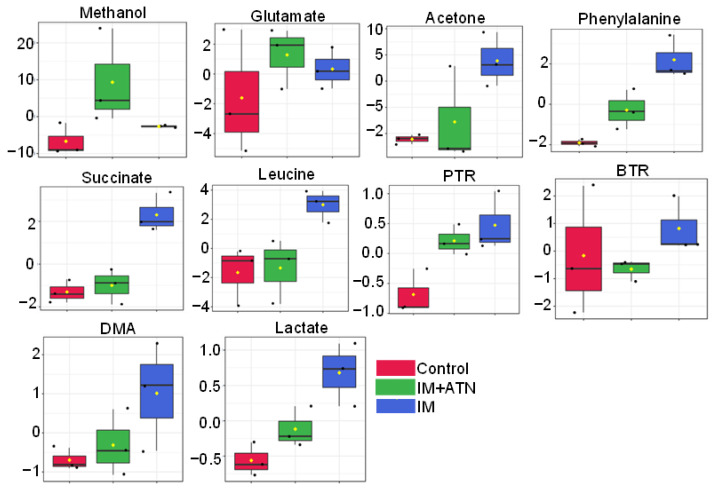
Representative box-cum-whisker plots showing quantitative variations in the concentrations of eleven serum metabolites. The black round dots along the *Y* axis in the box plots denote the concentrations of metabolites, while the yellow rhombus denotes mean concentrations of the group. In the box plots, boxes denote interquartile ranges, horizontal lines inside boxes denote medians, and the bottom and top boundaries of boxes represent the 25th and 75th percentiles, respectively. Lower and upper whiskers are 5th and 95th percentiles, respectively. Phenylalanine-to-tyrosine ratio (PTR), leucine-to-phenylalanine ratio (LPR), branch chain amino acid-to-tyrosine ratio (BTR), and dimethylamine (DMA). IM: Immobilized and ATN: Atenolol.

The 3D score plot derived by PLS-DA analysis revealed a clear separation between the three experimental groups, suggesting significant metabolic disparities in the IM and IM+ATN groups vs. the control group. Interestingly, the IM+ATN group showed a trend toward the control group as compared with the IM group (Figure S1A,B). The PLS-DA model validation parameters (Accuracy = 0.88, R^2^ = 0.91, and Q^2^ = 0.75) and predictive capability Q^2^ were high, suggesting significant metabolic differences between the three groups (Figure S2C). The metabolic features of discriminatory relevance were first identified by VIP score (>1.0) (Figure S1D). Univariate analysis was performed using ANOVA to determine the significances of intergroup differences. Univariate analysis (based on VIP and ANOVA) identified several metabolic entities capable of predicting therapeutic responses (Figure S1D), *viz.* (a) serum levels of acetone, glutamate, succinate, phenylalanine, leucine, serine, valine, isoleucine, citrate, dimethylamine (DMA), 3-hydroxybutyrate, ratios of BTR, QGR, and PTR were higher in the IM group than in the control group and (b) serum levels of alanine and proline were found to be lower in the IM group as compared to control group (Figure S1D and Table S2). Further, atenolol treatment recovered the metabolic changes in IM+ATN groups. Orthogonal projections to latent structure (OPLS-DA) models were used to differentiate two or more groups, and permutated analysis was used to check the robustness of the model. IM group, (C) IM+ATN vs. IM group, and (D) Control vs. IM+ATN group; (R2Y) = 0.435, 0.999, 0.998, and 0.988, respectively (Green bars) *p*-value < 0.05; and (Q2) = 0.182, 0.953, 0.943, and 0.612, respectively (Red bars) *p*-value < 0.05 (Figure S2A–D). These results show immobilization altered the levels of several key metabolites at play important roles in muscle wasting and that atenolol inhibited these changes. Quantitative variations in these discriminatory features are shown as box-cum-whisker plots in Figure 7.

## 4. Discussion

Several studies have been conducted to investigate the abilities of potential drugs/compounds to ameliorate muscle atrophy [11]. Our results show that atenolol intervention significantly reduced immobilization-induced muscle atrophy, as evidenced by the suppression of IM-induced changes in the levels of creatinine kinase, antioxidants (GSH, SOD, and catalase), and MDA (a marker of oxidative stress) and improved muscle coordination and cellular architecture. Prolonged bed rest reduces muscle size and strength in humans [36], and our results confirm that atenolol significantly reduces IM-induced body and GN muscle weight losses in immobilized rats, which support the results of previous studies which found that cast immobilization significantly reduces muscle weight in rats [37,38].

Furthermore, rotarod, forced swimming, and footprint analysis results showed that atenolol significantly inhibited IM-induced reductions in muscle coordination, stride length, and mobility. In addition, atenolol (10 mg/kg) shows improved ECG patterns and cardiac marker enzymes in the rat model of isoproterenol-induced myocardial infarction [39]. Additionally, atenolol (20 mg/kg) was administered to 5/6 nephrectomized rats, and this decreased cardiac fibrous tissue deposition, serum brain natriuretic peptide, and cardiac hypertrophy [40]. These findings demonstrated that atenolol may improve the functional and exercise capacity, and this could be one of the reasons to improve the behavioral parameters. Interestingly, atenolol (0.1 g L^−1^ in drinking water for 2–16 months) significantly decreased lipoxidative and glycoxidative damage in skeletal muscle and improved motor coordination in male B6D2F1 mice [17]. Furthermore, we found that atenolol treatment significantly suppressed IM-induced reductions in myofibrillar protein content, suggesting that atenolol might act via the anabolic pathway, which supports a report that skeletal muscle proteolysis is regulated by β2-adrenoceptors [41] and might be involved in protein synthesis. However, it was also reported that atenolol had no effect on epinephrine-induced degradation of soleus or extensor digitorum longus muscles [41]. Notably, it was also reported that immobilization increases the number of glucocorticoid receptors in the gastrocnemius [42]. Glucocorticoids bind to glucocorticoid receptor (GR) and increase the catabolism of muscle proteins via the ubiquitin–proteasome pathway. Interestingly, Sato et al. showed that cast-immobilization-induced muscle disuse reduces glucocorticoid receptors in slow-twitch muscle, which suggests that muscle disuse suppresses glucocorticoid signals and transcription of the β2-AR gene. The authors also reported no change in β2-AR protein levels after 10 days of cast immobilization [43]. These findings raise the possibility that atenolol might act via the glucocorticoid-receptor-mediated signaling pathway. However, it has also been reported that atenolol reduced angiotensin II levels in normotensive subjects and that angiotensin II levels are elevated in atrophic skeletal muscles [44,45]. Therefore, there is a possibility that atenolol may lower angiotensin II levels in immobilized muscle and that this explains how atenolol protects against muscle loss.

In addition, atenolol was found to improve IM-induced reduction in serum creatinine levels, which supports the notion that immobilization increased muscle permeability, resulting in creatinine leakage from muscles into the bloodstream. In a previous study, creatine supplementation attenuated cachexia and wasting-associated muscle loss [46,47], and in another, atenolol significantly suppressed immobilized-induced reductions in GSH, SOD, and catalase levels and increased MDA levels. Moreover, oxidative stress induces muscle atrophy via calpain activation, which increases protein degradation by up-regulating the proteasome pathway [48]. These results were supported by our histopathology findings. H&E staining revealed marked differences between the control and IM groups, and transverse sections of gastrocnemius showed that muscle fiber cross-sectional area and minimum Feret’s diameter were significantly higher in the IM+ATM group than in the IM group.

NMR-based serum metabolomics and multivariate analysis were used to investigate changes in metabolite levels in immobilized rats and the effects of atenolol. Serum levels of acetone, glutamate, succinate, phenylalanine, leucine, serine, valine, isoleucine, citrate, and dimethylamine and QGR and PTR were significantly higher in the IM group than in the control group, whereas LPR and proline levels were lower in the IM group. These results suggest that IM altered amino acid metabolism, which is essential for protein synthesis. Furthermore, increased levels of succinate (a TCA cycle intermediate) might disrupt mitochondrial membranes, increase inflammatory mediators and oxidative stress, and eventually cause muscle wasting. In addition, omega-3 supplementation ameliorates mitochondrial derangement in immobilization-induced muscle wasting in female subjects, suggesting that mitochondria functions are required for good muscle. We observed that homeostasis changes in TCA metabolites might contribute to muscle wasting [49]. We observed that rats in the IM group had higher levels of pyruvate and lactate than the control and atenolol inhibited these increases, suggesting a shift from fast twitch (type I) to slow twitch (type II) skeletal muscle fibers in gastrocnemius muscle. An IM-induced increase in fast myosin content and transition of slow to fast fibers were also reported in a rat model [50,51].

Atenolol also significantly suppressed IM-induced increases in serum phenylalanine, leucine, serine, valine, isoleucine, citrate, and dimethylamine metabolite levels, and these metabolites are key players in the TCA cycle, which showed that mitochondrial bioenergetics are required for proper function of mitochondria. Furthermore, higher serum PTR and QGR levels in immobilized rats indicate the presence of oxidative stress that might be responsible for the degradation of GN muscles and increased proteolysis. However, atenolol significantly reduced IM-induced increases in serum PTR and QGR levels, indicating that it reduced reactive species levels and suggesting that it has an antioxidant effect. This observation suggests that atenolol ameliorates the deleterious effect of cast immobilization in rats by reducing oxidative stress, improving antioxidant status, and preventing IM-induced changes in serum metabolites.

## 5. Conclusions

In conclusion, our findings suggest that atenolol reduces cast-immobilization-induced muscle atrophy and has potential use as a therapeutic intervention. Furthermore, the study demonstrates that serum metabolomics provide a powerful means of detecting muscle biomarkers in disease conditions.

## 6. Study Limitations

In the present study, we only studied the effects of immobilization and atenolol on rat gastrocnemius muscles after 14 days of cast immobilization. Furthermore, we did not explore the molecular mechanism involved, which, when elucidated, will undoubtedly provide an understanding of how atenolol suppresses immobilization-induced skeletal muscle loss.

## Data Availability

All data generated or analyzed during this study are included in this published article and available upon request.

## Supplementary Materials

The following supporting information can be downloaded at: https://www.mdpi.com/article/10.3390/biomedicines11051269/s1, Figure S1: Multivariate analysis; Figure S2: Internal validation of the corresponding OPLS-DA model by permutation analysis; Table S1: Identification of important serum prognostic metabolites in experimental groups (control, IM and IM+ATN); Table S2: Key prognostic metabolic differences between the serum analysis results of the control, IM and IM+ATN groups.
