# Supplementary material for: Atenolol Ameliorates Skeletal Muscle Atrophy and Oxidative Stress Induced by Cast Immobilization in Rats
Source: Biomedicines. 2023 Apr 25;11(5):1269. doi: 10.3390/biomedicines11051269 (PMC10215752; doi:10.3390/biomedicines11051269)
Supplement: Supplementary file 1 [file biomedicines-11-01269-s001.zip › biomedicines-2173027-supplementary.pdf]

Electronic Supplementary Material:

Atenolol Ameliorates Skeletal Muscle Atrophy and Oxidative Stress Induced by Cast Immobilization in Rats

Anand Kumar [1,†], Chaitany Jayprakash Raorane [2,†], Deepak Rawat [1], Priyanka Prajapati [1], Ritu Raj [3], Dinesh Kumar [3], Seong-Cheol Kim [2], Vinit Raj [2,*] and Sapana Kushwaha [4,*]

[1] Department of Pharmaceutical Sciences, School of Pharmaceutical Sciences, Babasaheb Bhimrao Ambedkar University, Vidya Vihar, Raebareli Road, Lucknow 226025, India
[2] School of Chemical Engineering, Yeungnam University, Gyeongsan 38541, Republic of Korea
[3] Centre of Biomedical Research, SGPGIMS Campus, Lucknow 226014, India
[4] National Institutes of Pharmaceutical Education and Research, Raebareli (NIPER-R), New Transit Campus, Bijnor-Sisendi Road, Delhi 226002, India
* Correspondence: drvinitraj@ynu.ac.kr (V.R.); sapana.k@niperraebareli.edu.in (S.K.)
† These authors contributed equally to this work.

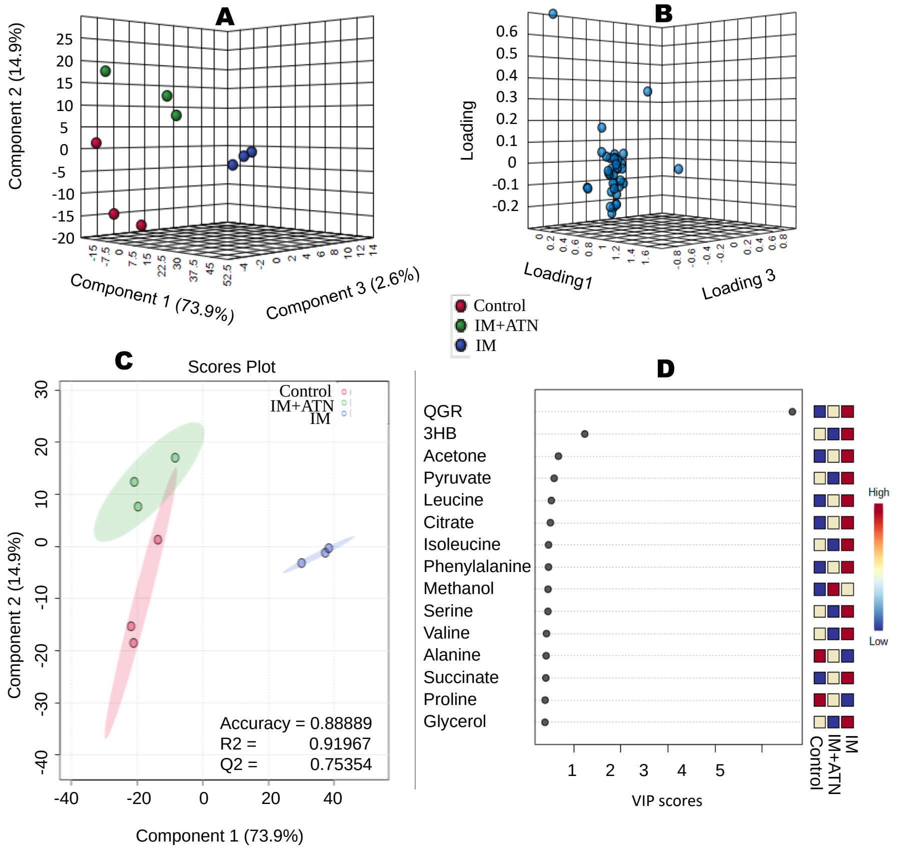

Figure S1. Multivariate analysis; **(A-B)** PLS-DA-based 3D score plot derived from the concentration profiles of serum metabolites. **(C)** PLS-DA based 2D score plot for for the separation of group trends **(D)** VIP score plot based on PLS-DA model shows indexing of metabolites based on their discriminatory potential shows important metabolic features exhibiting both discriminatory potential and statistically significant differences between the study groups. IM-Immobilized and ATN-Atenolol.

Electronic Supplementary Material:

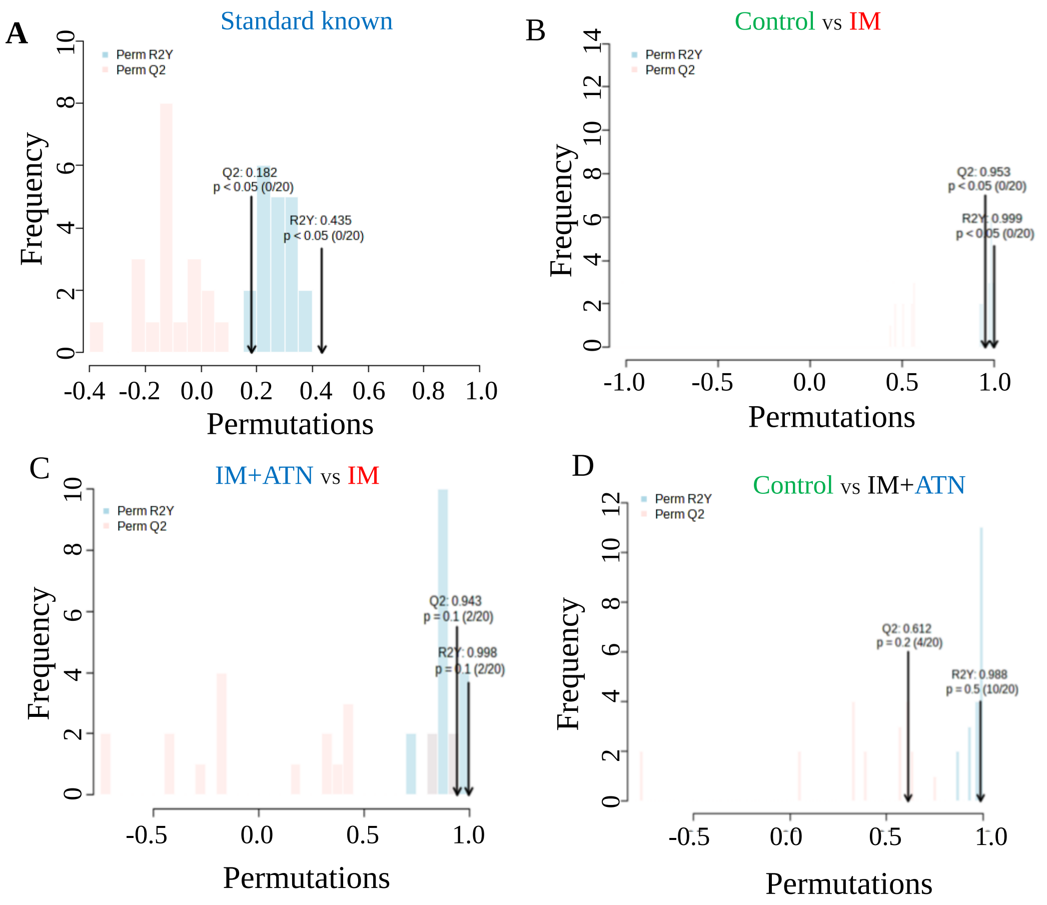

Figure S2. Internal validation of the corresponding OPLS-DA model by permutation analysis (n = 20); fraction of variance of descriptor class response (Y) and fraction of the variance predicted (cross-validated) (A) standard known, (B) Control *vs* IM group, (C) IM+ATN *vs* IM group, and (D) Control *vs* IM+ATN group; (R2Y) = 0.435, 0.999, 0.998, and 0.988 (Green bars) *p*-value < 0.05; and (Q2) = 0.182, 0.953, 0.943, and 0.612 (Red bars) *p*-value < 0.05. IM-Immobilized and ATN-Atenolol.

Electronic Supplementary Material:

Table S1. Identification of important serum prognostic metabolites in experimental groups (control, IM and IM +ATN). ^{1}H chemical shifts, variations, VIP scores, and p-values of individual biomarkers are given. *P*-values < 0.05 were considered significant. The metabolic differences between the IM and IM+ATN groups are shown. IM: Immobilized and ATN: Atenolol.

**Metabolites**	**^{1}H Chemical Shift**	**Variation in IM group with respect to control**	**VIP score**	**p-value**	**Variation in IM group with respect to IM + ATN**
3-hydroxybutyrate (3HB)	0.88, 1.55, 2.15	↑	1.417	0.972	↓
Acetate	1.92	↑	0.444	0.305	↓
Glutamine	2.35	↑	0.034	0.023	↓
Isobutyrate (IsoB)	1.073	↑	0.223	0.153	↓
Betaine	3.26, 3.91	↓	0.678	-0.465	↓
Proline	2.00, 2.06, 2.34, 3.34, 3.41, 4.13	↓	0.740	-0.508	↑
Choline	3.21	↓	0.756	-0.518	↑
Creatine	3.03, 3.93	↓	0.446	-0.306	
Alanine	1.48, 3.78	↓	0.847	-0.581	↑
Glucose	3.41, 3.54, 3.71, 3.73, 3.77, 3.83, 5.24	↓	1.117	-0.766	↑
Histidine to tyrosine ratio (HTR)		↑	1.209	0.829	↓
Leucine to phenylalanine ratio (LPR)		↓	0.685	-0.470	↑
Glutamate	2.11, 2.34, 3.76	↓	0.525	0.360	↑
Methanol	3.36	↓	0.911	0.625	↑
BCAA to tyrosine ratio (BTR)		↑	0.451	0.309	↓
Dimethyl sulphide (DMS)	2.65	↑	0.955	0.655	↓
Glycine	3.562	↑	0.853	0.585	↓
Myo-Inositol	3.628	↓	0.189	-0.130	↓
Glutamate to glutamine ratio (QGR)		↑	1.436	0.985	↓
Lactate	1.33, 4.18	↑	1.322	0.907	↓
Succinate	2.41	↑	1.372	0.941	↓
Dimethylamine (DMA)	2.72	↑	1.085	0.744	↓

Electronic Supplementary Material:

Table 2. Key prognostic

Phenylalanine to tyrosine ratio (PTR)		↑	1.257	0.862	↓
Phenylalanine	3.28, 3.99, 7.33,7.38, 7.43	↑	1.405	0.964	↓
Acetone	2.23	↑	1.363	0.935	↓
Leucine	0.96, 1.71, 3.73	↑	1.266	0.869	↓
Pyruvate	2.37	↑	1.368	0.939	↓
Serine	3.980	↑	1.007	0.691	↓
Isoleucine	0.93, 1.01, 1.25, 1.46, 1.97, 3.67	↑	1.328	0.911	↓
Valine	0.99, 1.04, 2.27, 3.61	↑	1.317	0.903	↓
Glycerol	3.563, 3.669, 3.794	↑	1.034	0.710	↓
Tyrosine	3.05, 3.19, 3.93, 6.86, 7.17	↑	1.121	0.769	↓
Citrate	2.55, 2.66	↑	1.391	0.955	↓
Threonine	1.33, 3.58, 4.25	↑	0.974	0.668	↓
Asparagine (ASN)	2.84, 2.94, 4.00	↑	0.116	0.080	↓
Histidine	3.11, 3.23, 3.97, 7.05, 7.77	↑	0.042	0.029	↓

Electronic Supplementary Material:

Table S2. Key prognostic metabolic differences between the serum analysis results of the control, IM and IM+ATN groups. ANOVA test, f- value, p-value, -log 10 (p), and false discovery rate (FDR) of individual metabolites are given. *P*-values < 0.05 were considered significant. Metabolic differences between the IM and IM+ATN groups are shown. IM: Immobilized and ATN: Atenolol.

**Metabolites**	**f-value**	**p-value**	**-log 10 (p)**	**FDR**	**Fisher's LSD**	
					**Variation in IM group with respect to control**	**Variation in IM group with respect to IM +ATN**
QGR	112.32	1.76E-05	4.7544	0.00065139	↑	↓
3HB	39.81	0.00034414	3.4633	0.0063	↑	↓
Pyruvate	34.529	0.00051081	3.2917	0.0063	↑	↓
Valine	27.18	0.00098226	3.0078	0.0090859	↑	↓
Succinate	19.711	0.002305	2.6373	0.017057	↑	↓
Phenylalanine	17.73	0.003031	2.5184	0.018691	↑	↓
Citrate	15.696	0.0041318	2.3839	0.021839	↑	↓
Isoleucine	14.836	0.0047586	2.3225	0.022009	↑	↓
Threonine	12.988	0.0066067	2.18	0.027161	↑	↓
Histidine	11.603	0.0086696	2.062	0.032078	↑	↓
Lactate	10.459	0.011074	1.9557	0.037249	↑	↓
